# Social Support–Seeking Strategies on Social Media at the Intersection of Lesbian, Gay, Bisexual, Transgender, and Queer Identity, Race, and Ethnicity: Insights for Intervention From a Qualitative Study

**DOI:** 10.2196/51702

**Published:** 2023-10-20

**Authors:** Jacob D Gordon, Darren L Whitfield, Tural Mammadli, César G Escobar-Viera

**Affiliations:** 1 Institute for Sexual and Gender Minority Health and Wellbeing Feinberg School of Medicine Northwestern University Chicago, IL United States; 2 School of Social Work University of Maryland Baltimore, MD United States; 3 Department of Psychiatry University of Pittsburgh Pittsburgh, PA United States

**Keywords:** intersectionality, LGBTQ+, minority stress, sexual and gender minorities, social media, social support, lesbian, gay, bisexual, transgender, and queer

## Abstract

**Background:**

Lesbian, gay, bisexual, transgender, and queer (LGBTQ+) individuals experience a disproportionately higher prevalence of mental health challenges when compared to their heterosexual and cisgender counterparts. Moreover, they exhibit increased engagement with social media platforms relative to their peers. Understanding the intersectional dynamics of their identities is crucial in elucidating effective and safe approaches to garnering social support through social media channels. This exploration holds significance for informing future research endeavors and shaping targeted interventions to address the unique mental health needs of LGBTQ+ individuals.

**Objective:**

The purpose of this study was to explore the strategies used by Black, Hispanic, and non-Hispanic White LGBTQ+ young adults to acquire social support from social media. The study aimed to examine how these strategies may differ by race and ethnicity.

**Methods:**

We conducted semistructured interviews with LGBTQ+ young adults aged between 18 and 30 years recruited in the United States from social media. Of 52 participants, 12 (23%) were Black, 12 (23%) were Hispanic, and 28 (54%) were non-Hispanic White. Thematic analysis was used to analyze the collected data.

**Results:**

The analysis uncovered both divergent and convergent strategies among participants of different races and ethnicities. Black and Hispanic young adults exhibited a preference for connecting with individuals who shared similar identities, seeking safety and tailored advice. Conversely, non-Hispanic White participants demonstrated minimal preference for identity-based advice. Seeking support from anonymous sources emerged as a strategy to avoid unwanted disclosure among Hispanic participants. Furthermore, all participants emphasized the importance of content filtering with family members to cultivate positive and supportive social media experiences.

**Conclusions:**

This study sheds light on the strategies used by LGBTQ+ individuals of different racial and ethnic backgrounds to seek social support from social media platforms. The findings underscore the importance of considering race and ethnicity when examining social support–seeking behaviors on social media in LGBTQ+ populations. The identified strategies provide valuable insights for the development of interventions that aim to leverage social support from social media to benefit the mental health of Black, Hispanic, and non-Hispanic White LGBTQ+ young adults.

## Introduction

Lesbian, gay, bisexual, transgender, and queer (LGBTQ+) individuals have a disproportionate burden of mental health concerns such as depression and anxiety [1,2]. Meyer’s [3] minority stress theory is the primary framework for explaining mental health disparities among LGBTQ+ people. Minority stress theory posits that identifying as an LGBTQ+ person increases stressors not experienced by cisgender and heterosexual peers, such as discrimination, social rejection, and isolation. These external stressors increase internalized homophobia and expectations of rejection, which in turn increase the risk for depression and anxiety among LGBTQ+ individuals [4]. Moreover, intersectionality theory describes how social categories or identities (eg, race, gender identity, and sexual orientation) intersect at the micro level to impact individual experiences (social media use, mental health, and substance use), which reflect interlocking systems of oppression and privilege at the macro level (eg, transphobia, homophobia, racism, and genderism) [5,6]. For example, Black and Hispanic LGBTQ+ individuals faced higher amounts of minority stress compared to non-Hispanic White LGBTQ+ individuals [7-9]. Minority stress and intersectionality contextualize the factors driving mental health disparities experienced by LGBTQ+ people of color [9,10]. These effects are moderated by social support from LGBTQ+ communities [11].

Social support refers to the emotional, informational, and instrumental help that people perceive from human interactions [12]. Social support is a strong protective factor against depression, anxiety, and other negative mental health outcomes for LGBTQ+ young adults [3,8,13]. For instance, LGBTQ+ individuals with high levels of minority stress show improved mental health outcomes if they perceive adequate social support [14]. One way that LGBTQ+ young adults seek effective social support is through social media [15-18]. Social media use is ubiquitous among LGBTQ+ persons to help connect with people of shared experiences [15,19], share grievances [20,21], and seek and obtain support to help cope with mental health concerns. Given that social media–based social support is easily accessible, independent of one’s physical space [15,22,23], there is potential for leveraging this source of social support for mental health prevention interventions among LGBTQ+ people. Indeed, previous qualitative research found that online connections can be an important source of support and affirming spaces for LGBT teens that mostly do not exist in their lives offline and that social media curation strategies may contribute to their well-being [24,25]. This led researchers to develop digital interventions to leverage online social support and improve mental well-being among LGBTQ+ youth and young adults [26].

While LGBTQ+ young adults might reap benefits from social support garnered from social media, seeking support online introduces the potential for negative interactions [27-29]. Moreover, youth are not always supportive of anonymous online spaces for promoting informal support and candid disclosures. Black, Hispanic, and non-Hispanic White LGBTQ+ young adults have to regularly navigate the identity disclosure processes when seeking social support on social media because often the stressors involve their own identities [30-32]. Online identity disclosure carries the risk of outing (eg, nonconsensual sharing of sexual or gender identities), harassment, and disclosure-related stress [31,33]. The potential of these unique risks for LGBTQ+ young adults suggests a highly nuanced social support–seeking process on social media. By exploring and comparing the social support processes of Black, Hispanic, and non-Hispanic White LGBTQ+ young adults on social media, we can further understand how they safely acquire social media–based social support.

Few studies have qualitatively explored the experiences and strategies of garnering social media–based social support among LGBTQ+ young adults [15,16], and no study has compared the strategies at the intersection of race and ethnicity among Black, Hispanic, and non-Hispanic White LGBTQ+ people. Based on the principles of intersectionality theory, current research has shown that the lived experiences of LGBTQ+ people of color differ significantly from those of White LGBTQ+ individuals. For instance, a qualitative study shows that LGBTQ+ people of color rarely experience discrimination based on their sexual orientation within LGBTQ communities. Instead, racism was prevalent within these communities [34]. This exploration is important because it will inform a more tailored development of prevention interventions for mental health that seek to leverage social support from social media among multiple marginalized groups. Thus, we sought to qualitatively compare the processes of eliciting social support on social media among Black, Hispanic, and non-Hispanic White LGBTQ+ young adults. Specifically, we (1) examined how LGBTQ+ young adults elicit social support on social media while navigating identity disclosure and (2) explored differences in the social support eliciting strategies based on racial and ethnic identities among LGBTQ+ young adults.

## Methods

### Recruitment

Data for this analysis came from a larger national study on the impact of social media experiences and behaviors on mental health among LGBTQ+ young adults; the full research protocol is described in detail elsewhere [35]. A summary of the procedures is included here. Recruitment was done through a purposive sampling approach to identify participants who experienced the phenomenon under study (ie, LGBTQ+ adults who are social media users) [36]. Study recruitment was done through social media (ie, Instagram and Facebook), which featured targeted advertisements. The advertisements were set to be shown to only people living in the United States and were specifically set up to target interest tags related to the LGBTQ+ community. To be selected in this study, individuals had to be aged between 18 and 30 years, identify as LGBTQ+, live inside the United States, have a minimum of one social media profile, and use it on a weekly basis. Eligible individuals provided informed consent before the start of each individual web-based interview. Between July 2019 and February 2020, participants completed a 60-minute web-based semistructured interview on various aspects of social media interactions, including the management of their social media presence and seeking and obtaining social support on the web. Interviews were conducted by CGEV and 2 trained graduate students. A total of 52 participants completed the interviews. Participants were compensated for their time. We obtained verbatim transcriptions using a professional service.

### Analysis

Sociodemographic analysis was performed to characterize the sample. To address the primary research questions of this study, we used a reflexive deductive thematic analysis approach on 2 open-ended questions included in the interviews: “Do you choose to connect with people of similar or different identities?” and “Tell me about your experience getting social support from social media.” Qualitative thematic content analysis is appropriate for larger data sets, as it lends itself to a team-based approach with multiple coders working to develop consensus [37]. The analytic process involved a progression from description to interpretation of thematic patterns [37]. For this study, this included an iterative process of familiarizing data, generating primary and subcodes, reviewing codes, creating higher-order themes, and theme-defining. Analysis was done first independently among all authors, but consensus on meanings and themes was reached among the diverse analysis team in a second meeting [37]. Lastly, the 4 analysts independently coded the transcripts and met for a final time to discuss how the codes were applied. The Cohen κ coefficient was calculated for each pair of analysts (κ=0.71, κ=0.73;) using NVivo (version 12; QSR International), which was used to assign codes, manage, and sort the data.

### Ethics Approval

All recruitment and data collection procedures were approved by the University of Pittsburgh Institutional Review Board (study 19050007; approval June 20, 2019). The original informed consent allows the secondary analysis without additional consent for this study. All data are deidentified. Participants received online gift cards in compensation for their time taking part in each of the research activities. Given the inclusion of sensitive topics and questions in the interview, we informed participants that they could choose to stop taking part at any time if they felt uncomfortable. Participants were given US $50 gift cards for their participation.

## Results

### Participant Characteristics

In total, 52 LGBTQ+ young adults completed the interviews. Participants were aged between 18 and 30 years (mean 25.6, SD 3.9). The sample consisted of Black (12/52, 23%), Hispanic (12/52, 23%), and non-Hispanic White (28/52, 54%) LGBTQ+ young adults. Further, of these participants, 38% (20/52) of individuals identified as transgender and 62% (32/52) as cisgender. Participants reported high levels of use among multiple social media applications, including 79% (41/52) Instagram, 65% (34/52) Facebook, and 52% (27/52) YouTube. Table 1 shows sample characteristics.

**Table 1:** Sample characteristics (N=52).

Sociodemographic groups and usage			Participants, n (%)
**Sociodemographic groups**			
	**Race and ethnicity**		
		Black	12 (23)
		Hispanic	12 (23)
		Non-Hispanic White	28 (54)
	**Gender identity**		
		Transgender	20 (38)
		Cisgender man	16 (31)
		Cisgender woman	16 (31)
	**Sexual orientation**		
		Bisexual	18 (35)
		Gay	15 (29)
		Lesbian	6 (12)
		Pansexual	6 (12)
		Queer	7 (13)
	**Residence**		
		Urban	35 (67)
		Rural	17 (33)
	**Highest educational attainment**		
		High school graduate	8 (15)
		Some college	20 (38)
		College graduate	9 (17)
		Currently in graduate school	15 (29)
**Social media usage**			
	**Facebook use**		
		High use	34 (65)
		Low use	8 (15)
		No use	12 (23)
	**Twitter use**		
		High use	17 (33)
		Low use	10 (19)
		No use	25 (48)
	**Reddit**		
		High use	8 (15)
		Low use	1 (2)
		No use	43 (83)
	**YouTube**		
		High use	27 (52)
		Low use	17 (33)
		No use	8 (15)
	**Instagram**		
		High use	41 (79)
		Low use	4 (7)
		No use	7 (13)
	**Snapchat**		
		High use	23 (44)
		Low use	6 (12)
		No use	23 (44)

### Themes

#### Overview of Themes

The following 3 overarching themes emerged based on the interpretation of the coding team: (1) sources of support with similar identities, (2) use of mutually anonymous support from strangers, and (3) strategies to restrict family presence on social media. Table 2 presents a summary of the primary themes and subthemes, which are described in detail in the following sections. Each theme emerged from the analysis of the combined responses from both open-ended questions. Taken together, the individual questions contributed equal amounts to each theme.

**Table 2:** Overview of themes.

Number	Primary themes	Subthemes
1	Sources of support with similar identities	Safe environment Tailored advice Nonprioritization of shared identities
2	Use of mutually anonymous support from strangers	Psychological safety Passive support
3	Strategies to restrict family presence on social media	Avoiding conflict Finding context-specific advice

#### Theme 1: Sources of Support With Similar Identities

Participants’ social identities played a significant role in the process of seeking online social support from social media. Specifically, Black and Hispanic participants indicated that their sexual orientation, race or ethnicity, and gender identity influenced how they sought social support from others using social media. In contrast, non-Hispanic White participants had little preference for similar identities and, in some cases, preferred receiving social support from those who shared no identities with them.

#### Safe Environment

Black and Hispanic participants felt safer when seeking online social support from others that shared similar sexual, gender, and racial or ethnic identities. Black and Hispanic participants spoke to the importance of avoiding racial tensions, even amongst other LGBTQ+ young adults, when seeking social support. For instance, one respondent mentioned the following:

In some of the larger LGBT groups that I’m a part of, there’s a lot of racial tensions. So, when I have these conversations, I try and mostly have them with other LGBT people of color.

Black, cisgender man, gay

Several participants mentioned that feeling safe among those with similar identities was a precursor to their seeking social support. A respondent explained this process in the context of sexual orientation identity:

I think knowing that someone is gay allows for a safer connection and environment to go and ask for help.

Hispanic, cisgender man, gay

#### Tailored Advice

Participants shared that one benefit of social support elicited from those with similar identities is that the social support is more relevant to their lived experience. For example, one participant revealed that advice given by a heterosexual individual was not transferable to the life that they lived.

I remember when I didn’t have gay friends. I would confide in a straight friend that I was out to. And they would help, but it wouldn’t be 100%. I mean I appreciate it…but some of the advice looking back was not completely transferrable between straight and LGBT.

Hispanic, cisgender man, gay

Participants expressed that tailored advice was more trustworthy and addressed specific challenges that may have been unknown to other individuals with different identities. Black and Hispanic interviewees mentioned the importance of shared intersectional identities (ie, race or ethnicity, sexual orientation, and gender identity):

The people providing that support have mainly been transgender individuals or gender non-conforming people. And it’s been like, some of the things that they’ve said that they’ve experienced, and I can kind of see myself in that. That’s been pretty helpful in terms of like how to deal with things.

Hispanic, transgender man, gay

#### Nonprioritization of Shared Identities

No specific preference toward shared identities emerged from non-Hispanic White participants when seeking social support on social media. Some mentioned that there was a risk of creating an “echo chamber” (ie, a lack of diversity of thought) if they only included those who looked like them:

I am concerned of being in an echo chamber. Occasionally, I’ll find and keep people that I differ with if they’re constructive in sharing their viewpoints.

non-Hispanic White, cisgender man, gay

Several participants highlighted the value of social support from individuals who differed from them, emphasizing the significance of diverse perspectives. One respondent reflected on this perspective:

I don’t care about that (their identity). I don’t care if somebody’s gay, bi and straight. I just want people to be themselves.

non-Hispanic White cisgender woman, lesbian

#### Theme 2: Use of Mutually Anonymous Support From Strangers

Some participants sought support from strangers whose identities were unknown to the respondents using social media. While shared social identities are a crucial factor in seeking social support on the web for some Black and Hispanic LGBTQ+ young adults in this sample, a large number of Hispanic interviewees emphasized the significance of anonymity when seeking social support through social media. This emphasis on anonymity might stem from heightened feelings of psychological safety and the ease of accessing passive support. Interestingly, non-Hispanic White and Black participants either explicitly expressed their aversion to using anonymous sources of support or did not mention them at all. For instance, one participant mentioned:

It’s never been strangers. It has always been friends who answered or who would send me a message.

Black, cisgender man, gay

#### Psychological Safety

One key factor that was mentioned by Hispanic participants was the psychological safety that came with anonymous sources of support. Interviewees mentioned that eliciting social support from strangers was less risky psychologically. For instance, one participant reflected that they did not internalize negative critiques of their identities when disclosing their identities to strangers:

So, it’s not really a critique of you if somebody doesn’t support you, it’s a critique of whatever that comment happened to be. So, I came out to strangers before I came out to people that I knew.

Hispanic, transgender man, bisexual

Moreover, interviewees also shared that using anonymous sources of support made them feel less of a burden, which motivated them to seek social support while avoiding placing any undue strain on their nonanonymous social network, namely their family and friends, as reported by this respondent:

Sometimes it’s easier when you don’t know the person because if you go to a person that you do know, you might worry that they’re going to worry for you too much. You don’t want to burden them with your problems or how you’re feeling.

Hispanic, transgender man, gay

#### Passive Support

Interviewees adopted a passive approach when using anonymous sources of support. Several respondents specifically referred to Reddit, a popular social media platform in the United States, as a notable outlet for this form of passive anonymous support:

Reddit especially has helped me to connect with people. Sometimes it’s better for me to connect with people I don’t know personally. I’ve gone to just read other people’s posts to find people who are thinking similar to me.

Hispanic transgender man, gay

By engaging with relevant posts that mirrored their own experiences as LGBTQ+ young adults, participants found solace and support without having to comment or disclose their personal identities. This enabled them to seek and receive social support while maintaining anonymity. One respondent reported:

I go to Reddit for support, and I don’t really make any comments. It’s just me scrolling through what other people have experienced and a lot of the times some of those experiences align with the ones that I’ve had. It’s been really great to see how they’ve dealt with it.

Hispanic, cisgender man, gay

#### Theme 3: Restricting Family Presence on Social Media

LGBTQ+ participants described different strategies to manage their presence on social media. This included limiting the number of individuals who could view content related to their identity and maintaining different accounts based on their identities. When seeking social support through social media, Black, Hispanic, and non-Hispanic White LGBTQ+ participants expressed preferences for filtering social support content from familial members on social media. Restricting their social support content from their family on social media offered a range of reported benefits, such as being able to manage their identity disclosure risk, discovering spaces that fostered authenticity, and receiving tailored advice from others who shared their lived experience.

#### Avoiding Conflict

Interviewees filtered their own social media content that elicited social support from family members. This provided interviewees with a sense of safety, allowing them to avoid conflicts, judgment, and the need for self-censorship. One respondent reflected on this process:

But the family.... Ironically, they’re the ones that usually argue the most any time I post something. So sometimes I filter them out. I change the settings on who sees it, and occasionally if I think, “Okay, there’s going to be a fight about this,” I will hide it from certain family members.

non-Hispanic White, cisgender man, gay

While specific filtering methods varied, participants also reported actions such as blocking family members from accessing certain posts and using family-free social media apps. These measures empowered participants to maintain control over their social media interactions and seek support in a secure environment, as reported by this respondent:

I add my family, but I have to block them off my posts on Facebook because I don’t want to see their reactions. They act like they gotta comment or talk about something and it’s just like, leave me alone. This is who I am and I don’t want you to judge me or bother me based on what I post.

Black, cisgender man, gay

#### Finding Context-Specific Advice

Participants expressed concerns regarding the limited ability of their family members to provide relevant and effective social support for the unique challenges they encountered due to their marginalized identities. Interviewees found that families were not a source of useful social support due to them not understanding their intersectional identities. One respondent reflects:

I love my family very much and they’ve helped me a lot through a lot of my [gender] transition. But the fact is that they inherently don’t understand where a lot of my mental issues come from.

non-Hispanic White, transgender man, queer

Similar to previous strategies, participants took measures such as blocking family members from accessing specific social support posts and using social media apps that prevented family connections. These actions were aimed at creating curated spaces where participants could freely express their authentic selves and receive tailored advice from other LGBTQ+ young adults. One participant mentioned:

I mean family is fine, but my family doesn’t fully understand who I am, so I get more meaningful advice from my friends that know who I am

Black, nonbinary, bisexual

## Discussion

### Principal Results

The primary objective of this qualitative examination was to gain insight into the different strategies for seeking social media–based social support among LGBTQ+ young adults at the intersection of race and ethnicity. Existing literature highlights the significance of social support in buffering the effects of minority stressors for LGBTQ+ young adults; however, there is a noticeable lack of research considering how one’s racial or ethnic identity may influence the process of seeking social support on social media [18,35]. A total of 3 themes emerged: sources of support with similar identities, use of mutually anonymous support from strangers, and restricting family presence on social media. Our findings highlight the importance of considering the preferences of LGBTQ+ people of color when developing digital interventions that seek to leverage social media–based social support for mental health among marginalized groups.

#### Sources of Support With Similar Identities

Participants described the importance of shared social identities when seeking individuals and platforms for social support on social media. Compared to White participants, Black and Hispanic LGBTQ+ persons described the value of receiving social support on social media from individuals who shared their multiple identities.

These findings are consistent with and expand existing research on social support–seeking among LGBTQ+ people of color by highlighting the unique experience of Black and Hispanic LGBTQ+ people when navigating social media for social support [23,38,39]. Seeking social support from others who have also experienced unique intersectional minority stressors may be vital for coping with minority stress. Our findings suggest the coping strategy of seeking social support from others of multiple, shared intersectional social identities offline also extends to social media. Black and Hispanic LGBTQ+ young adults’ preference for social support from other individuals with the same identities is likely due to their ability to relate to their challenges and offer guidance based on their own experiences of heterosexism, homophobia, racism, xenophobia, and transphobia [40].

Non-Hispanic White interviewees reported seeking online social support from those with differing identities, expressing a desire for individuals who did not resemble themselves to refrain from creating an “echo chamber” on their social media platforms. This finding is a new addition to current literature that explores preferences for echo chambers on social media among LGBTQ+ people [41]. Non-Hispanic White participants were less concerned about the intersectional identities of the people on their social media. In comparison, Black and Hispanic respondents sought social support from those with similar identities, expressing that they felt safer or could avoid conflict. One explanation for these findings might be that time spent on social media has been linked to depression and anxiety among Black and Hispanic adolescents and young adults through racial discrimination [42,43], and higher levels of negative affect during active engagement on social media were observed among Black youth, potentially due to online discrimination [44]. Thus, Hispanic and Black LGBTQ+ young adults may seek similar identities when eliciting social support to avoid online discrimination on social media. Future mental health prevention intervention research aimed at leveraging social media to gather social support for LGBTQ+ people should recognize the significance of intersecting identities as a crucial component for effective support among Black and Hispanic LGBTQ+ young adults.

#### Use of Mutually Anonymous Support From Strangers

In this study, Hispanic participants expressed a preference for anonymous forms of social support. These findings align with and expand the limited body of literature that indicates transgender adolescents were likely to seek mutually anonymous social support compared to other forms of support on the web [15]. While Hispanic LGBTQ+ people preferred anonymity when seeking social support from social media, Black and non-Hispanic White participants did not express interest in seeking support from anonymous sources. This could stem from cultural and ethnic differences in managing identity disclosure.

One rationale for seeking anonymous support among Hispanic individuals may be related to cultural differences. Hispanic LGBTQ+ individuals navigate identity disclosure in unique ways compared to non-Hispanic LGBTQ+ young adults, as previous research suggests that values of familism (eg, the belief that family must remain physically and emotionally close when possible) and machismo (eg, a social attitude that values masculinity) influence the coming-out process [45-47]. Contrary to masculine norms, Hispanic LGBTQ+ individuals publicly seeking social support or identifying as gay or bisexual on the web may increase the risk of family rejection. This may influence their preference for anonymous forms of support. Additionally, limited literature also suggests that the levels of familism and machismo are related to factors such as generation in the United States (ie, first or second generation in the United States) and migration status [48]. Understanding why Hispanic LGBTQ+ young adults prefer anonymity when seeking social support becomes crucial when taking an intersectional perspective that considers both migration and generation status. While previous research found that youth and young adults do not always support the idea of gathering online social support from anonymous sources [32], intervention development must consider the cultural and ethnic variations in individuals’ perceptions of identity disclosure risks that might influence their preference for anonymous sources of social support in the future.

#### Filtering and Management of Social Media Profiles Restricting Family Presence on Social Media

Across racial and ethnic identities, interviewees discussed several similar strategies for managing and filtering their social media content to maximize opportunities for social support. Participants compartmentalized their social media presence to ensure that accidental disclosure of their identities did not occur with their family members. These findings complement and expand existing research on how LGBTQ+ youth curate their social media presence to ensure they do not encounter harassment or victimization [25,49,50]. LGBTQ+ young adults created specific social media personas, filtered content, and blocked family members to secure social support. These findings suggest that social media platforms where individuals feel more comfortable disclosing their identity might be more suitable for digital interventions that seek to increase perceived social support among LGBTQ+ people. While these could be platforms specifically developed for LGBTQ+ communities, previous research indicates that leveraging already existing platforms and providing information and resources about platform features that facilitate content filtering and profile management has better chances of user engagement and intervention usage [51]. However, these coping strategies may mask individuals’ authentic selves and hinder genuine connection when seeking social support on the web. There is limited literature indicating that self-disclosure on social media is a crucial pathway for effective social support, and this intricate process of filtering and avoiding identity disclosure may in fact reduce the likelihood of obtaining social support as originally intended [52,53]. Future research should examine the psychological impact of filtering and masking one’s identities and how LGBTQ+ young adults include or exclude family when seeking social support from social media interactions.

### Implications

Our findings support the use of social media platforms that enhance the likelihood of marginalized identity disclosure, which is a crucial element of effective social support for LGBTQ+ individuals. Digital interventions aimed at bolstering social support for LGBTQ+ individuals through social media should consider the unique strategies used by LGBTQ+ individuals from diverse racial and ethnic backgrounds. Beyond intervention research, our results underscore the potential benefits of social media in addressing identity-related stressors. When making blanket policy decisions concerning the restriction of social media access for both youth and adults, it is important to consider the potential of social media use as a means of obtaining identity-specific social support for LGBTQ+ individuals.

### Strengths, Limitations, and Future Directions

This study has limitations to consider. The open-ended questions used for our analysis may introduce social desirability bias among participants; however, our interview guide was developed in a way to avoid including leading statements or questions that would influence their response. Despite a large sample for an exploratory and qualitative study, we did not examine variations in factors such as depressive symptoms, discrimination, and outness to family. There is research to suggest that how individuals use social media and to what extent it may be effective for social support may depend on their current levels of depressive symptomatology [44]. Further, this study did not examine differences by age. Adolescent and teenage LGBTQ+ people may have particular preferences in their social support–seeking behavior on social media. Our recruitment methods are exclusively focused on social media and might exclude individuals who are not on social media. However, our research questions were heavily focused on social media experiences, so recruiting social media users was appropriate. Future research should explore how Black and Hispanic LGBTQ+ teenagers navigate social media-based social support based on these factors. Additionally, we did not examine how social media platforms and the strategies used by users may evolve over time. While one-time qualitative assessments served the purpose of exploring these strategies at a single point in time, future qualitative studies might want to consider longitudinal or pre-post designs that could better serve to explore change in users’ strategies over time. Despite these limitations, this study engaged a high number of Black, Hispanic, and non-Hispanic White LGBTQ+ individuals, which allowed for a detailed comparison of social support strategies.

### Conclusions

We examined the diverse social support–seeking strategies of Black, Hispanic, and non-Hispanic White LGBTQ+ young adults on various social media platforms. The strategies used by LGBTQ+ people of color differ considerably based on their personal identities and levels of outness on social media. Hispanic respondents display a preference for anonymous sources of social support, likely to mitigate identity disclosure risks. In contrast, non-Hispanic White respondents show less preference for individuals with similar identities compared to both Black and Hispanic participants. Our findings confirm that interventions leveraging social media for social support should consider the potential of the different preferences of Black, Hispanic, and non-Hispanic White LGBTQ+ young adults. Additionally, the impact of ongoing identity disclosure processes on the social support–seeking process should be considered, particularly among LGBTQ+ young adults’ family members. Researchers should consider the need for tailored approaches rather than a one-size-fits-all approach to enhance social support acquired through social media among LGBTQ+ young adults.

## Abbreviations

LGBTQ+: lesbian, gay, bisexual, transgender, and queer

## Data Availability

The data sets generated during and/or analyzed during this study are available from the corresponding author on reasonable request.
